# Reactive Oxygen Species from NADPH Oxidase and Mitochondria Participate in the Proliferation of Aortic Smooth Muscle Cells from a Model of Metabolic Syndrome

**DOI:** 10.1155/2018/5835072

**Published:** 2018-12-23

**Authors:** Ocarol López-Acosta, María de los Angeles Fortis-Barrera, Miguel Angel Barrios-Maya, Angélica Ruiz Ramírez, Francisco Javier Alarcón Aguilar, Mohammed El-Hafidi

**Affiliations:** ^1^Universidad Autónoma Metropolitana Unidad Iztapalapa, Doctorado Ciencias Biológicas y de la Salud, Avenida San Rafael Atlixco 186, Vicentina, Iztapalapa, 09340 Ciudad de México, Mexico; ^2^Instituto Nacional de Cardiología Ignacio Chávez, Departamento de Biomedicina Cardiovascular, Juan Badiano No 1, Colonia Sección XVI, Tlalpan, 14080 Ciudad de México, Mexico; ^3^Universidad Autónoma Metropolitana Unidad Iztapalapa, Laboratorio de Farmacología, Departamento de Ciencias de la Salud, Avenida San Rafael Atlixco 186, Vicentina, Iztapalapa, 09340 Ciudad de México, Mexico

## Abstract

In metabolic diseases, the increased reactive oxygen species (ROS) represents one of the pathogenic mechanisms for vascular disease probably by promoting vascular smooth muscle cell (SMC) proliferation that contributes to the development of arterial remodeling and stenosis, hypertension, and atherosclerosis. Therefore, this work was undertaken to evaluate the participation of ROS from NADPH oxidase and mitochondria in the proliferation of SMCs from the aorta in a model of metabolic syndrome induced by sucrose feeding in rats. After 24 weeks, sucrose-fed (SF) rats develop hypertension, intra-abdominal obesity, hyperinsulinemia, and hyperleptinemia. In addition SMCs from SF rats had a higher growth rate and produce more ROS than control cells. The treatment of SMCs with DPI and apocynin to inhibit NADPH oxidase and with tempol to scavenge superoxide anion significantly blocked the proliferation of both SF and control cells suggesting the participation of NADPH oxidase as a source of superoxide anion. MitoTEMPO, which targets mitochondria within the cell, also significantly inhibited the proliferation of SMCs having a greater effect on cells from SF than from the control aorta. The higher rate of cell growth from the SF aorta is supported by the increased content of cyclophilin A and CD147, proteins involved in the mechanism of cell proliferation. In addition, caldesmon, *α*-actin, and phosphorylated myosin light chain, contractile phenotype proteins, were found significantly lower in SF cells in no confluent state and increased in confluent state but without difference between both cell types. Our results suggest that ROS from NADPH oxidase and mitochondria significantly participate in the difference found in the rate of cell growth between SF and control cells.

## 1. Introduction

Vascular smooth muscle cells (SMCs) under physiological conditions present a differentiated contractile/quiescent phenotype, characterized by a considerably low proliferation range and low synthetic activity of the extracellular matrix and by the expression of signaling molecules for cell contractile functions [1, 2]. The SMC proliferative phenotype may be characterized by a decreased content of contractile proteins such as *α*-actin, phosphorylated myosin light chain, and caldesmon and by an increased production of extracellular matrix proteins that contribute to the development of arterial stenosis, arterial remodeling, hypertension, and atherosclerosis, among other pathologies [3–5]. In cardiovascular tissue, several physiological processes such as the differentiation, proliferation, and apoptosis involve ROS generation. Mitochondria and NADPH oxidase are considered as the main sources of ROS in cardiovascular tissue and are involved in different physiological and pathological processes depending on the intensity of ROS generation [6–9]. Under physiological conditions, ROS levels are found in a steady state which is defined by the generation and elimination of ROS. In pathological conditions, this steady-state ROS concentration is transiently or chronically increased and results in intermediate-intensity oxidative stress or high-intensity oxidative stress which are associated with cellular metabolism alteration or with cellular component damage allowing cell death by apoptosis or necrosis [10–13]. In intermediate-intensity oxidative stress, ROS were described to play a role as a second messenger to influence signal transduction and to reprogram general cellular functions by upregulating the expression of antioxidant enzymes and certain genes of cell proliferation [13]. Indeed, it was described that ROS-induced vascular SMC proliferation is mediated by the secretion of cyclophilin A (CyPA) which acts through its receptor CD147 and activates JAK-ERK1/2-Akt signaling pathways to enhance DNA biosynthesis and proliferation of SMCs [7, 14, 15]. This mechanism of ROS-induced oxidative stress and SMC proliferation may be considered as a mechanism involved in the development of cardiovascular disease in metabolic diseases such as abdominal obesity, insulin resistance, and hypertension [16, 17]. It is well known that obesity promotes the release of several factors such as cytokines and lipotoxic free fatty acids (FFAs) that interact and accumulate in the cell and affect mitochondrial function and NADPH oxidase activity to increase ROS generation [18–21]. In this context, ROS by enhancing cell dysfunction and altering vascular SMC phenotype and cell environment may contribute to the activation of mechanisms that promote vascular SMC proliferation and migration or death. Thus, the study of SMC proliferation from a model of intra-abdominal obesity induced by sucrose feeding, in where NADPH oxidase activity and oxidative stress markers were found increased in vascular tissue, is of great interest to elucidate the mechanisms of metabolic syndrome (MS) inducing cardiovascular diseases and involving the participation of ROS generation. Moreover, the model develops resistance to insulin, hypertension, and alterations in vascular reactivity by increasing vasoconstriction in response to phenylephrine and reduced vasorelaxation in response to acetylcholine [22] and this makes it useful to investigate the mechanism by which SMC proliferation is a risk factor of cardiovascular disease development in metabolic diseases. Thus, the use of SMCs extracted from the aorta of this model to elucidate the mechanism by which ROS contributes to SMC proliferation and their altered contractile proteins *in vitro* represents a relevant approach for understanding the *in vivo* ROS action. In addition, there is no data about the behavior of SMC in this model of abdominal obesity induced by sucrose nor about the participation of mitochondria or NADPH oxidase in ROS generation and SMC proliferation. As NADPH oxidase, mitochondria are considered as the main source of ROS such as superoxide anion (O_2_^·−^) generated by leak of electrons from the redox centers of respiratory complexes I and III to molecular oxygen [23]. In this model of obesity induced by sucrose diet, we also reported several metabolism alterations such as high circulating FFA and oxidative stress associated with mitochondrial ROS generation in the liver [24]. Therefore, the objective of this research was to investigate the participation of mitochondria and NADPH oxidase as sources of ROS on SMC proliferation, the protein profile of contractile phenotype, and cell signaling involving CyPA in a model of central obesity induced by high-sucrose diet.

## 2. Materials and Methods

### 2.1. Experimental Animals

Newly weaned male Wistar rats weighing 55 ± 5 g were used. Animals were obtained from the animal facility of the National Institute of Cardiology Ignacio Chávez and were processed according to the National Institutes of Health *Guide for the Care and Use of Laboratory Animals*. The animals were divided into groups of 6 rats each: the control groups (C) received solid food (Lab Diet Formula 5001, Ralston Purina Corp., St. Louis, MO) and water ad libitum. The sucrose-fed (SF) groups received a solution of sucrose (refined sugar) at 30% as drinking water and the same solid food ad libitum. After 24 weeks of treatment, rats were fasted overnight and the next day they were sacrificed by cervical dislocation. After 24 weeks of sucrose treatment, systolic and diastolic blood pressure was measured as described previously [25].

### 2.2. Leptin, Insulin, FFA, TG, and Glucose

The blood was collected from the abdominal aorta into tubes containing anticoagulant (0.1% EDTA) and immediately centrifuged at 600 ×g for 20 min at 4°C. To plasma thus obtained, 0.005% of butylated hydroxytoluene (BHT) was added as an antioxidant and the mixture was stored at −70°C until analysis. FFAs were determined by gas chromatography as described previously [25] and were obtained according to the method of Folch et al. [26]. TG plasma concentration was measured according to the method described by Nägele et al. [27]. Glucose levels, plasma insulin, and leptin were measured by radioimmunoassay using standard commercial kits (Linco Research). The intra-abdominal fat was dissected off retroperitoneal cavity and around both kidneys and weighed immediately. Visceral and duodenal fat was not included in this procedure.

### 2.3. Primary SMC Culture

SMCs were obtained from the aorta under sterile conditions. The aorta was collected and immediately placed in a buffer containing 140 mM NaCl, 4.7 mM KCl, 1.2 mM Na_2_HPO_4_, 2.4 mM MgSO_4_, 2 mM CaCl_2_, 5.6 mM glucose, 0.02 mM EDTA, 25 mM HEPES, and pH 7.4 and fat was removed. In a first step, the defatted aorta was incubated with 1 mg/ml of collagenase type II (125 U/mg activity, Gibco) for 20 min to remove the adventitia (fibroblasts) from smooth muscle. The second step consisted of incubating the aorta without adventitia for 40 min with 10 mM papain (30 U/mg activity, Roche) to disaggregate the cells. Subsequently, the obtained suspension was filtered through a 230 *μ*m pore sieve (Tissue Grinder Kit, Sigma) and seeded in 25 cm^2^ cell culture flasks (Corning) in D-MEM/F-12 culture medium (Gibco) supplemented with 10% fetal bovine serum (FBS) (Gibco) inactivated at 56°C for 30 min. SMCs were grown at 37°C with 5% CO2 and 90% humidity. When the cells reached confluent state, they were treated with 0.25% trypsin-EDTA (Gibco) and reseeded in a 75 cm^2^ cell culture flask, in the same culture medium described above [5].

When cells reach a confluent state in the 75 cm^2^ flasks, they were treated with 0.25% trypsin-EDTA (Gibco) and reseeded in multiwell cell culture plates (24 wells) at 8000 cells per well in the medium containing 10% FBS and the medium was renewed every 24 h. After washing, SMCs were finally fixed at different times 0, 24, 48, 72, 96, and 120 h with absolute ethanol to determine DNA quantity. DNA was determined according to the modified method of Brunk et al. [28] using 4′,6*-*diamidino*-2-*phenylindole (DAPI) as a fluorescent probe at 360 nm (excitation wavelength) and 450 nm (emission wavelength) using a Perkin Elmer LS50B spectrofluorometer. Cells fixed in ethanol were treated with 0.150 ml of NaOH (1 N) and 1.35 ml of 0.15 M Na_2_HPO_4_ containing DAPI at a final concentration of 0.5 *μ*M. To further evaluate the amount of DNA in samples, a curve was performed with DNA extracted from salmon (Sigma) whose concentration of the stoke solution was adjusted with NanoDrop (Thermo Scientific).

### 2.4. SMC ROS Generation

Cells were grown on coverslips treated with poly-L-lysine (0.1%). Once cells were adherent, they were preincubated with 5 *μ*M of 2′,7′diclorodihidrofluorescein diacetate (DCF-DA) for 15 min and then washed with PBS to remove excess DCF. Cells were washed again with PBS and mounted on slides with glycerol. The oxidized DCF was detected by confocal microscopy.

### 2.5. Mitochondria as a Source of ROS

SMCs were incubated with MitoSOX (5 *μ*M) for 15 min. MitoSOX is accumulated selectively in mitochondria and it emits red fluorescence when oxidized by superoxide anion. Then, cells were fixed with 1% paraformaldehyde for 30 min at 4°C. Finally, cells were mounted on coverslips with glycerol for analysis by confocal microscopy.

### 2.6. ROS Participation in SMC Proliferation

SMCs were seeded under the same conditions described above in 24 multiwell plates in the absence and presence of hydroxy-TEMPO (tempol) and MitoTEMPO, superoxide anion scavengers, in cytosol and mitochondria, respectively, and in the presence of diphenyleneiodonium chloride (DPI) and apocynin (APO), NADPH oxidase inhibitors. All inhibitors were tested at different concentrations, from 1 to 10 *μ*M for 72 and 120 h to assess the participation of ROS in cellular proliferation and which sources of ROS are contributing to this process.

### 2.7. Contractile Phenotype Protein Markers

Proteins were extracted from SMCs in two proliferative states: in exponential growth state (72 h) and in the confluent state (120 h). At the end of experiments in different conditions, cells were harvested and lysed in a buffer containing 25 mM HEPES, 0.1 M NaCl, 15 mM imidazole, 10% glycerol, 1% Triton X-100, protease inhibitors (115 mM phenylmethylsulfonyl fluoride (PMSF), 2 mM leupeptin, 1.5 mM aprotinin, and 3 mM pepstatin), and antiphosphatases (0.2 mM orthovanadate, 50 mM NaF).

The suspension was then incubated for 30 min at 4°C. At the end of incubation time, the sample was centrifuged at 8000 ×g for 10 min at 4°C to remove cell debris. The homogenate protein level was quantified using the method of Bradford [29]. Two hundred micrograms of protein of each sample was collected, suspended in 25 *μ*l of buffer load containing 125 mM Tris-HCl (pH 6.8), 20% glycerol, 4% SDS, 10% 2-mercaptoethanol, and 0.004% bromophenol blue, and completed to 50 *μ*l with Laemmli solution (40 mM Tris, 1% SDS, and 1% *β*-mercaptoethanol).

Eighty micrograms of protein of each sample was loaded in SDS-PAGE gel which acrylamide percentage depended on the protein to analyze. The electrophoresis was run for 3 h at 120 V. The protein transfer was performed onto a nitrocellulose membrane (pore size 0.22 *μ*m, Bio-Rad) at 350 mA for 60 min for caldesmon, CD147, 45 min for *α*-actin, ERK 1/2 and GAPDH, and 35 min for phosphorylated myosin light chain and CyPA in a chamber of semidry transfer (Bio-Rad, Trans-Blot SD). The nonspecific protein detection was reduced by blocking membranes with TBS (25 mM Tris base, 150 mM NaCl) containing 5% nonfat milk and 0.1% Tween 20. Then, the membranes were incubated with monoclonal antibodies against anti-caldesmon (Abcam, 1 : 20000), anti-alpha actin (Abcam, 1: 300), antiphosphorylated myosin light chain (p-MLC) (Abcam, 1: 1000), anti-CyPA (Abcam 1 : 500), anti-CD147 (Abcam, 1: 2000), anti GAPDH (Abcam, 1: 10000), anti-ERK 1/2 (GeneTex, 1 : 1000), and anti-beta actin as a load control (Novusbio, 1 : 10000). The secondary antibodies used were peroxidase conjugated. Proteins were revealed by chemiluminescent reagent (Immobilon, Millipore), and membranes were exposed to radiography plates (BioMax, Kodak) for 5 min. The image was scanned with an imaging system GelDoc-It (UVP Inc., Upland, CA, USA). Bands were analyzed by a UVP image analyzer, and optical density was evaluated with the VisionWorks LS software (UVP Inc., Upland, CA, USA).

### 2.8. Statistical Analysis

Statistical analysis was performed using the SigmaPlot program (version 11). Data are presented as the mean + SE (standard error). The number of experiments varied depending of the type of experiment (*n* = 4 to 8). One-way ANOVA was used for comparing the data from different groups. The difference between groups was considered statistically significant when *p* < 0.05.

## 3. Results

### 3.1. General Characteristics of Animals

The treatment of rats with sucrose diet for 24 weeks induced a statistically significant increase in heart rate and diastolic and systolic blood pressure (*p* < 0.01). In addition, triglycerides, FFA, insulin, and leptin in plasma and intra-abdominal fat tissue were found higher in SF rats (*p* < 0.01). On the other hand, analysis of total cholesterol and glucose and body weight showed no significant difference between the two groups. Cholesterol associated with HDL decreased significantly in SF animals (*p* < 0.05) (Table 1).

### 3.2. Smooth Muscle Cell Proliferation

**Figure 1:**
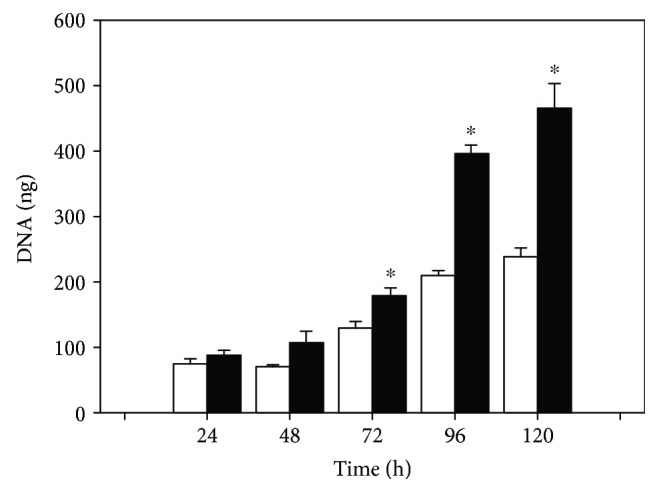
The proliferation of SMCs from C rats (open bars) and SF (black bars) in the presence of 10% SFB was determined by quantifying DNA using DAPI (0.5 *μ*M) as a fluorescent probe. The SMCs were cultured at different growth times 24, 48, 72, 96, and 120 h. The values correspond to the medium of the amount of DNA (ng) ± SE (*n* = 6 independent experiments and each experiment corresponds to one animal). ^∗^*p* < 0.05 corresponds to C vs. SF.

Figure 1 shows that the amount of DNA corresponding to SMCs from SF rats increased significantly across the time as compared to control cells and at any time of cell growth (*p* < 0.05). After 24, 48, 72, 96, and 120 h of cell seeding, DNA amount corresponding to SMCs from SF animals increased by 18%, 55%, 40%, 89%, and 95%, respectively, as compared with SMCs from control animals. The increase of DNA over time reflected a greater proliferation of SMCs isolated from the SF model than that from the control animals.

**Figure 2:**
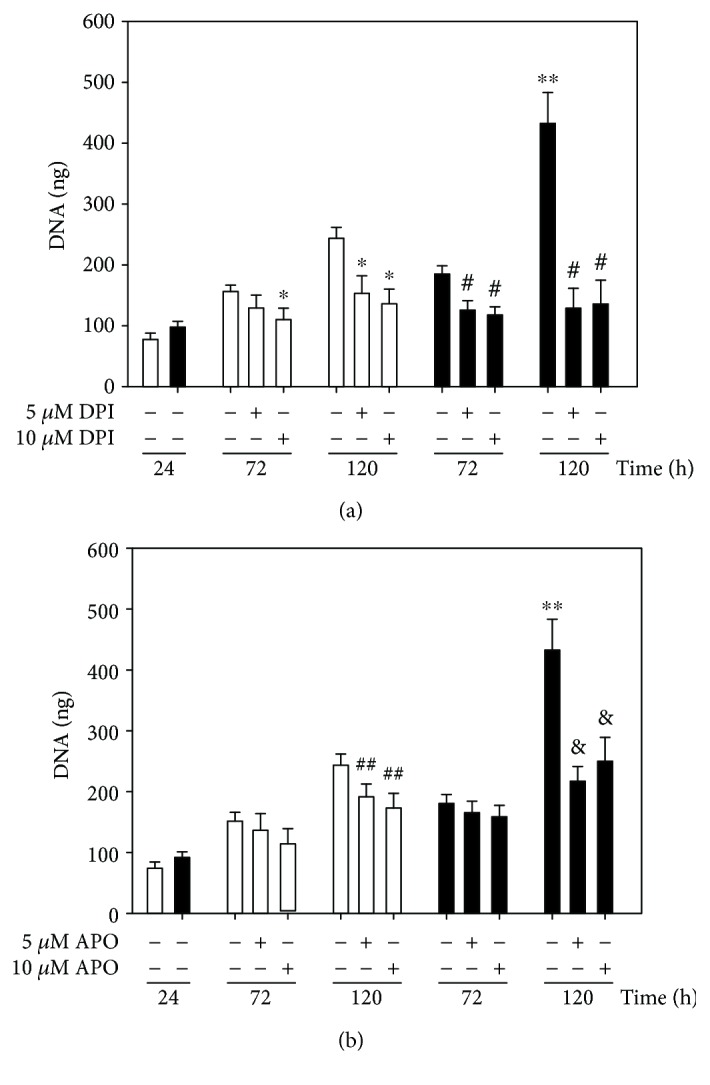
Effect of DPI (a) and APO (b) on the proliferation of SMCs from control rats (open bars) and SF (black bars) cultivated during 72 and 120 h to 5 and 10 *μ*M of each compound. The values correspond to the average of the amount of DNA (ng) ± SE (*n* = 3 independent experiments and each experiment corresponds to one animal). ^∗∗^*p* < 0.05 C vs. SF, ^∗^*p* < 0.05 C vs. C + DPI, ^#^*p* < 0.05 SF vs. SF + DPI, ^##^*p* < 0.05 C vs. C + APO, and ^&^*p* < 0.05 SF vs. SF + APO.

**Figure 3:**
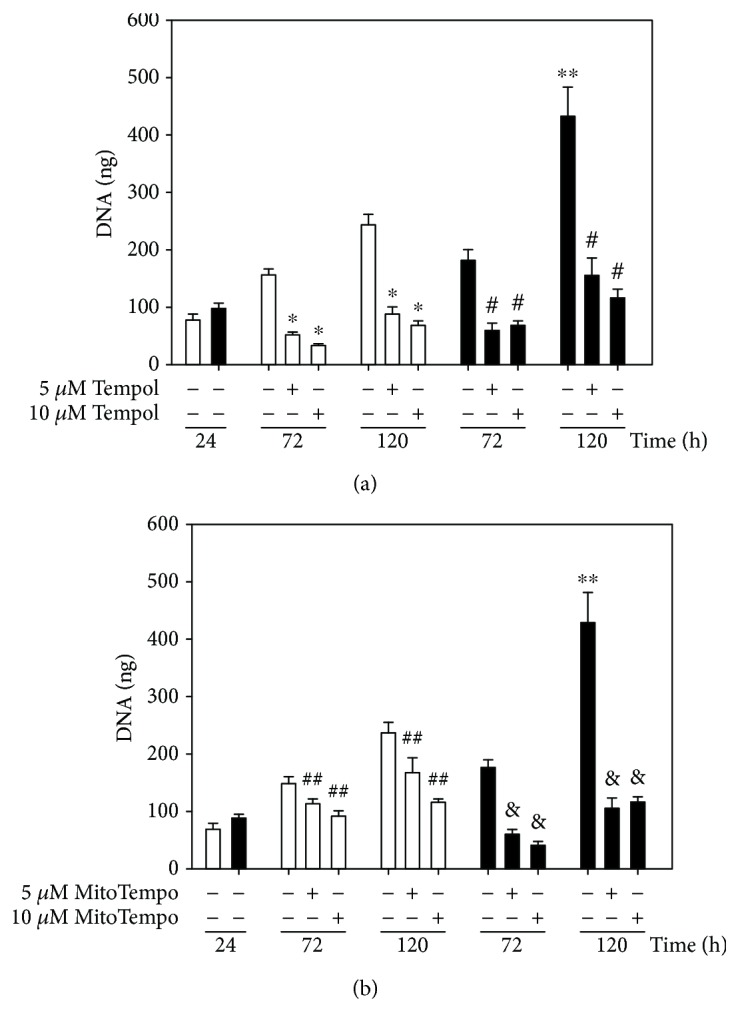
Effect of tempol (a) and MitoTEMPO (b) on SMC proliferation of control rats (open bars) and SF (black bars) cultured during 72 and 120 h to 5 and 10 *μ*M of each compound. The values correspond to the amount of DNA (ng) ± SE (*n* = 3 independent experiments). ^∗∗^*p* < 0.05 C vs. SF, ^∗^*p* < 0.05 C vs. C+tempol, ^#^*p* < 0.05 SF vs. SD+tempol, ^##^*p* < 0.05 C vs. C + MitoTEMPO, and ^&^*p* < 0.05 SF vs. SF + MitoTEMPO.

In order to elucidate the involvement of ROS generation in SMC proliferation, several inhibitors of ROS sources within cell were used. Apocynin (APO) and DPI were used as inhibitors or NADPH oxidase (Figure 2), while tempol was used as a superoxide anion scavenger. Moreover, MitoTEMPO, a superoxide radical scavenger at the mitochondrial level, was used to elucidate the participation of mitochondria in ROS-induced cell proliferation (Figure 3).

With DPI at 5 *μ*M, a proliferation-blocking effect by 32% was observed in SF cells at 72 h (Figure 2(a)). At 120 h, both at 5 and 10 *μ*M, DPI decreased DNA in cells from SF control animals (by 70% and 40%, respectively). In the case of apocynin, Figure 2(b) shows that the proliferation of SMCs from the SF aorta was blocked by 45% at 120 h. When SMCs were incubated with tempol, a significant inhibition of cell growth (*p* < 0.05) was observed at 72 and 120 hours by 70% in control SMCs and by 65% in SF cells (Figure 3(a)). When SMCs were incubated with MitoTEMPO that selectively targets mitochondria, a greater inhibitory effect was observed on the proliferation of SMCs isolated from the SF aorta by 70% and in control SMCs by 30% (Figure 3(b)). SMCs from SF rats were more sensitive to the proliferation-inhibiting effect of MitoTEMPO than control cells at both 72 and 120 h of culture.

### 3.3. Smooth Muscle Cell ROS Generation

**Figure 4:**
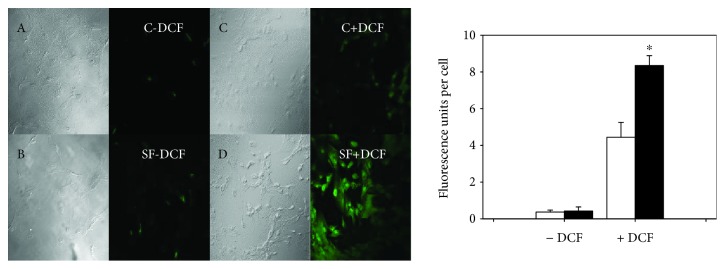
Generation of ROS at cell wall level in SMCs of control rats (open bars) and with metabolic syndrome (black bars) analyzed by confocal microscopy. The values correspond to the intensity of the fluorescence emitted by cell ± SE (*n* = 3 independent experiments). A, B, C, and D correspond to the images of optic microscopy of C-DCF, SF-DCF, C + DCF, and SF + DCF, respectively. ^∗^*p* < 0.05 correspond to C vs. SF.

**Figure 5:**
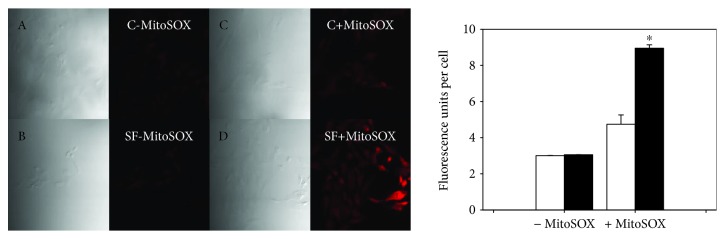
Generation of ROS at the mitochondrial level in SMCs of control rats (open bars) and with metabolic syndrome (black bars) analyzed by confocal microscopy. The values correspond to the intensity of the fluorescence emitted by cell ± SE (*n* = 3 independent experiments). A, B, C, and D correspond to the images of optic microscopy of C-DCF, SF-DCF, C+DCF, and SF+DCF, respectively. ∗ corresponds to C vs. SF (*p* < 0.05).

MitoSOX and DCF, two ROS probes, were used to monitor ROS generation at mitochondria and cell wall levels, respectively.  Figure 4 shows a significant increase (*p* < 0.05) of DCF fluorescence in SF cells by 46% compared with control cells. To evaluate ROS generation from SMCs at mitochondria level, MitoSOX, a fluorogenic dye specifically targeted to mitochondria, was used. It emits red fluorescence when oxidized by superoxide. In microscopy images (Figure 5), both control and SF primary cultured SMCs without MitoSOX emitted intrinsic fluorescence. When cells were incubated with MitoSOX, the intensity of fluorescence was 48% significantly more intense in cells from SF model compared with cells from control animals (*p* < 0.05).

### 3.4. Analysis of Cyclophilin A, Cyclophilin A Receptor (CD147), and ERK-1/2

**Figure 6:**
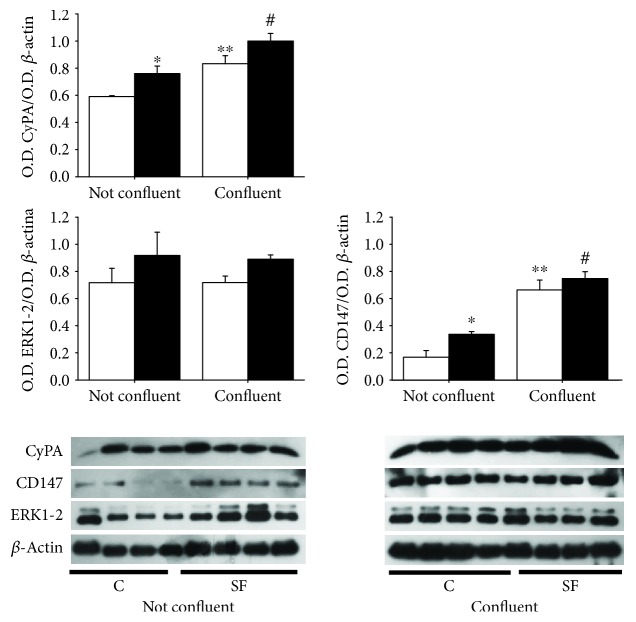
Analysis by western blot of cyclophilin A (CyPA: 17 kDa), cyclophilin A receptor (CD147: 55 kDa), and ERK1-2 (46 kDa) in SMCs (C, open bars; SF, black bars) during nonconfluent growth state (72 h) and confluent state (120 h). The values in the graphs correspond to the ratio of optic density (O.D.) CyPA/O.D. *β*-actin, O.D. CD147/O.D. *β*-actin, and O.D. ERK1-2/O.D. *β*-actin ± SE (*n* = 4 different animals). ^∗^*p* < 0.05 C not confluent vs. SF not confluent, ^∗∗^*p* < 0.05 C not confluent vs. C confluent, and ^#^*p* < 0.05 SF not confluent vs. SF confluent.

Figure 6 shows the expression of cyclophilin A whose content was 28% higher under nonconfluent conditions in SF cells compared to control cells. When cells reach confluence, the content of this protein increased in both SF and control cells by 32% and by 40%, respectively, in comparison with nonconfluent control and SF cells. However, the difference between both cell types is lost in confluent state.

In regard to CD147 under nonconfluent conditions, a significant increase (*p* < 0.05) in SF cells was observed compared to control cells, but at confluent state, the content of this protein was enhanced in both cell types and there was no difference between SF and control cells (Figure 6).

With respect to ERK 1/2, the content was the same for SF and control cells at nonconfluent conditions and confluent conditions (Figure 6).

### 3.5. Contractile Phenotype Protein

**Figure 7:**
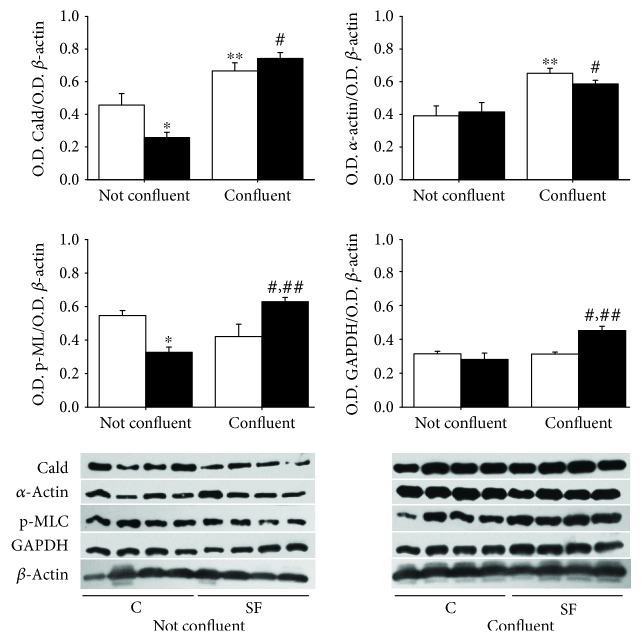
Content analysis of caldesmon (Cald: 70 kDa), *α*-actin (37 kDa), phosphorylated myosin light chain (p-MLC: 50 kDa), and GAPDH (37 kDa) quotient in SMCs (C, open bars; SF, black bars) during nonconfluent growth state (72 h) and confluent state (120 h). The values in the graph correspond to the ratio of O.D. Cald/O.D. *β*-actin, O.D. *α*-actin/O.D. *β*-actin, O.D. p-MLC/O.D. *β*-actin, and O.D. GAPDH/O.D. *β*-actin ± SE (*n* = 4 different animals). ^∗^*p* < 0.05 C not confluent vs. SF not confluent, ^∗∗^*p* < 0.05 C not confluent vs. C confluent, ^#^*p* < 0.05 SF not confluent vs. SF confluent, and ^##^*p* < 0.05 C confluent vs. SF confluent.

SMCs are well characterized by the expression of several contractile phenotype proteins, such as caldesmon, *α*-actin, and the phosphorylated myosin light chain (p-MLC). Their abundance may depend on the cell growth state. Thus, nonconfluent (72 h) and confluent (120 h) growth conditions were tested to evaluate the expression of these proteins.  Figure 7 shows that the content of caldesmon was lower under conditions of nonconfluence compared to that in the confluent state, in both cell types. Moreover, the O.D. corresponding to caldesmon band level at 72 h was significantly lower in SMCs from the SF aorta compared to control cells (*p* < 0.05). This difference between SF and control cells was lost at 120 h. In regard to *α*-actin content, the O.D. analysis shows a significant increase in its content when cells were in confluence as compared with growing state (72 h) and no difference was observed between SF and C cells (Figure 7). The content of p-MLC in nonconfluent state was significantly lower in SMCs from SF compared to that from control rats (*p* < 0.05). In confluent state, the distribution of p-MLC was inverted and the amount of p-MLC was shown enhanced in SMCs from SF animals as compared with control cells (Figure 7). The GAPDH tended to be lower in both control and SF cells in nonconfluent state compared with confluent cells. In addition, GAPDH content increased in the isolated SMCs of the SF model in confluent conditions (Figure 7).

## 4. Discussion

This study was undertaken to investigate the role of endogenous ROS in the contractile and proliferative phenotype of SMCs isolated from SF model, since it is reported that a high-sucrose diet increases the generation of ROS in different tissues including vascular tissue [24, 30]. The proliferation and migration of SMCs is a physiological process in response to pathological conditions such as metabolic syndrome associated with hyperglycemia and hyperlipidemia, hypertension, and diabetes [31]. All these pathological alterations are characterized by oxidative stress due to the enhanced steady-state concentration of ROS. When the basal concentrations of ROS increases and exceeds antioxidant defense, it also may induce alterations of intracellular redox status by the modification of GSH/GSSG and NAD(P)/NAD(P)H involved in the interaction between signaling protein (docking) and in the activation or depression of several proteins involved in cell proliferation and cell death [13, 32]. Our results of metabolic alterations induced by sucrose feeding, such as high adipose tissue, hypertriglyceridemia, hyperleptinemia, and hyperinsulinemia, are associated with increased diastolic and systolic blood pressure and heart rate and may contribute to the generation of ROS [33]. The participation of ROS in cell proliferation is evidenced by incubating cells with different scavengers and inhibitors of ROS sources. As an inhibitor of NADPH oxidase, DPI acts differentially from APO and it decreases the amount of DNA in both SF and control cell types at 72 and 120 h, having a greater effect on SF cells, suggesting a cytolytic effect. In addition to its wide use as an inhibitor of NADPH oxidase, DPI has been reported to inhibit other important enzymes such as NADPH ubiquinone oxidoreductase, nitric oxide synthase, xanthine oxidase, NADPH cytochrome P450 oxidoreductase, and cholinesterase that may be involved in cell survival [34]. Apocynin however is a specific inhibitor of NADPH oxidase [35]. It acts more specifically by inhibiting the association of p47phox and of p67phox with gp91phox as NADPH oxidase subunits, without affecting other proteins that may be vital for cell survival [36, 37]. In our work, it significantly blocks cell growth without causing a decrease in DNA concentration in SF suggesting that there is no cytolytic effect and the participation of NADPH oxidase in the proliferation of SMCs from the SF aorta. In regard to the use of tempol as a selective scavenger of superoxide anion and permeable to biological membranes [38], the results suggest the participation of superoxide anion generated at different levels such as NADPH oxidase and mitochondria.

Unlike tempol, MitoTEMPO selectively targets mitochondria because of its chemical structure having triphenyl phosphonium as a lipophilic cationic functional group [39]; its selective inhibitory effect on SF cell proliferation indicates that the superoxide radical generated at mitochondria level also plays a very important role in the proliferation of SMCs from SF rats. Indeed, the experiments of ROS generation at mitochondrial level, using MitoSOX that accumulates in the mitochondria and that reacts specifically with superoxide anion, indicate an increase in superoxide anion generation by SMCs isolated from SF rats compared to control cells.

In a previous study, mitochondria isolated from the liver of animals with sucrose diet generated ROS at high rate probably due to lipid metabolic alterations such as TG and FFA found increased in SF rats [24]. In SMCs from the SF aorta, ROS may be a modulating signal for growth factors, by regulating transcription factors that control the expression of genes associated with proliferation [8, 40]. SMC proliferation associated with ROS release has been described to be related to the secretion of several proteins called SOFX (secreted oxidative stress-induced factors), among them CyPA. CyPA secretion involves several proteins that participate in a cytoskeleton remodeling process and in the formation of vesicles necessary for CyPA secretion [41]. CyPA upon binding to its CD147 receptor overexpressed in SMCs from the SF aorta may stimulate JACK/Akt/ERK1/2 pathways and promote cell proliferation [7, 42].

In this work, the amount of CyPA found higher in isolated cells from SF model in growing state (nonconfluent) indicates the increased availability of CyPA to be secreted out of cell and to interact with CD147 also found increased to induce ERK1/2 expression and activity involved in DNA biosynthesis. The direct involvement of ROS in CyPA expression and secretion was not addressed. However, the blocking cell proliferation by inhibiting ROS generation in both mitochondria and NADPH oxidase suggests further experiments to evidence the ROS involvement in CyPA, CD147, and ERK1/2 expression activity and their relation to cell proliferation. Indeed, the increased CyPA expression and secretion was described to be blocked by antioxidants in Nox1-transfected cells [14].

In regard to the SMC contractile phenotype characterized by the expression of proteins such as *α*-actin, caldesmon, and phosphorylated myosin light chain [43–45], our results suggest that SMCs did lose partially the contractile phenotype during the growth state (not confluent), and when the cells reach confluent state, they totally recover the contractile phenotype. These results suggest that the method using papain for enzymatic dispersion of SMCs from the rat aorta keeps cell with a contractile phenotype. However, the lower content of caldesmon and p-MLC in nonconfluent SF cells could be related with a decrease in contractile phenotype of SMCs from the SF aorta, higher ROS generation, and lipotoxicity in SF animals. The finding of increased p-MLC in confluent state of SMCs isolated from SF may be related to the increased vasoconstriction found in aorta ring and associated with the hypertension that characterizes SF rats [46, 47].

In summary, SMCs from SF model have a higher proliferation range due to a higher generation of superoxide anion at the level of NADPH oxidase and mitochondria. In addition, ROS favors the expression and probably the secretion of cyclophilin A that may act through its CD147 receptor to promote SF SMC proliferation, decreasing contractile phenotype proteins. As a consequence of the finding of ROS involvement in cell proliferation suggests to implement of strategies using ROS generation inhibitors to control SMC proliferation in SF rats.

## Figures and Tables

**Table 1:** General characteristics of animals.

Variables	C	SF
Body weight (g)	488.0 ± 19.1	477.0 ± 31.7
Heart rate (beat/min)	351.9 ± 5.2	371.9 ± 2.2^∗∗^
Systolic blood pressure (mmHg)	111.7 ± 2.3	130.2 ± 2.9^∗∗^
Diastolic blood pressure (mmHg)	69.0 ± 2.7	97.8 ± 3.9^∗∗^
Medium blood tension (mmHg)	82.8 ± 2.4	108.7 ± 3.4^∗∗^
Intra-abdominal fat (g)	7.4 ± 2.2	15.3 ± 2.6 ^∗^
TGs (mM)	0.8 ± 0.1	1.8 ± 0.1 ^∗∗^
Glucose (mM)	5.9 ± 0.1	5.9 ± 0.2
Total cholesterol (mM)	1.5 ± 0.1	1.4 ± 0.1
Cholesterol-HDL (mg/dl)	42.65 ± 3.5	28.1 ± 4.1^∗^
FFAs (mM)	0.6 ± 0.1	1.1 ± 0.1 ^∗∗^
Insulin (pM)	99.6 ± 5.1	167.6 ± 7.8^∗∗^
Leptin (ng/ml)	0.6 ± 0.2	2.7 ± 0.3^∗∗^

The values were expressed as a medium + SE (*n* = 7 different animals). The values of all the variables were obtained at the end of the treatment period (24 weeks). ^∗^*p* < 0.05 and ^∗∗^*p* < 0.01 correspond to SF vs. C.

## Data Availability

The data used to support the findings of this study are available from the corresponding author upon request.

## Abbreviations

APO: Apocynin
BHT: Butylated hydroxytoluene
Cald: Caldesmon
C: Control
CyPA: Cyclophilin A
DAPI: 4′,6*-*Diamidino*-*2*-*phenylindole
DCF-DA: 2′,7′-Dichlorodihydrofluorescein diacetate
DPI: Diphenyleneiodonium chloride
FFAs: Free fatty acids
FBS: Fetal bovine serum
NADPH: Nicotinamide adenine dinucleotide 2′-phosphate reduced
MS: Metabolic syndrome
PMSF: Phenylmethylsulfonyl fluoride
p-MLC: Phosphorylated myosin light chain
ROS: Reactive oxygen species
SMCs: Smooth muscle cells
SF: Sucrose-fed
Tempol: 4-Hydroxy-2,2,6,6-tetramethylpiperidine 1-oxyl
TG: Triglycerides.
